# Measurement of Physical Activity and Energy Expenditure in Wheelchair Users: Methods, Considerations and Future Directions

**DOI:** 10.1186/s40798-017-0077-0

**Published:** 2017-03-01

**Authors:** Tom E. Nightingale, Peter C. Rouse, Dylan Thompson, James L. J. Bilzon

**Affiliations:** Department for Health, University of Bath, Bath, BA2 7AY UK

## Abstract

Accurately measuring physical activity and energy expenditure in persons with chronic physical disabilities who use wheelchairs is a considerable and ongoing challenge. Quantifying various free-living lifestyle behaviours in this group is at present restricted by our understanding of appropriate measurement tools and analytical techniques. This review provides a detailed evaluation of the currently available measurement tools used to predict physical activity and energy expenditure in persons who use wheelchairs. It also outlines numerous considerations specific to this population and suggests suitable future directions for the field. Of the existing three self-report methods utilised in this population, the 3-day Physical Activity Recall Assessment for People with Spinal Cord Injury (PARA-SCI) telephone interview demonstrates the best reliability and validity. However, the complexity of interview administration and potential for recall bias are notable limitations. Objective measurement tools, which overcome such considerations, have been validated using controlled laboratory protocols. These have consistently demonstrated the arm or wrist as the most suitable anatomical location to wear accelerometers. Yet, more complex data analysis methodologies may be necessary to further improve energy expenditure prediction for more intricate movements or behaviours. Multi-sensor devices that incorporate physiological signals and acceleration have recently been adapted for persons who use wheelchairs. Population specific algorithms offer considerable improvements in energy expenditure prediction accuracy. This review highlights the progress in the field and aims to encourage the wider scientific community to develop innovative solutions to accurately quantify physical activity in this population.

## Key Points

- Predicting energy expenditure from physical activity is inherently more challenging in persons with chronic physical disabilities who use wheelchairs due to altered movement patterns and variations in metabolically active muscle mass.
- Recent studies have successfully attempted to utilise technological advancements (i.e. multi-sensor devices) to measure physical activity and predict energy expenditure (using population or activity-specific algorithms) in persons who use wheelchairs.
- Combining measurement methods (both self-report and objective) might provide greater contextual information about the types and purpose of activities being performed by persons who use wheelchairs. This has implications to inform the wider public health agenda by promoting physical activity and reducing non-communicable diseases in persons with chronic physical disabilities.

## Review

### Introduction

Considerable evidence now exists to support the beneficial effects of physical activity (PA) for human health and wellbeing [1–3]. However, the majority of this evidence is from research in adults without disabilities. Our understanding of the impact and importance of PA for populations with chronic physical disabilities, particularly those who use wheelchairs, is therefore lacking. One of the major barriers to the acquisition and analysis of PA data in populations who use wheelchairs is the uncertainty surrounding the validity and reliability of the existing PA measurement tools. Improved assessment of habitual PA would permit; appropriate cross-sectional comparisons to biomarkers of metabolic health allow researchers to comment on the efficacy of behaviour change interventions and potentially inform PA guidelines [4]. This review provides a detailed evaluation of the available tools within the context of their potential application in persons who use wheelchairs. Firstly, three of the most frequently utilised self-reported measures will be described and evaluated. Then our attention will turn to the increasingly employed objective methods of measuring PA.

### Population Considerations

The PA behaviour of persons who use wheelchairs is inherently difficult to measure due to the heterogeneous nature of the population, whereby different disability aetiologies responsible for the use of a wheelchair result in highly variable movement patterns. Common physical disabilities that require prolonged use of a wheelchair include spinal cord injury (SCI), amputation, multiple sclerosis, cerebral palsy and cerebrovascular disease. Currently, it is problematic to accurately equate PA into units of energy expenditure (EE), as EE varies significantly from person to person depending on body mass, type of physical disability and efficiency of movement. Due to movement being primarily restricted to the upper body, the energy cost of most exercise and activities of daily living performed by persons who use wheelchairs result in a considerably lower energy cost (−27%) than those reported in the general population [5, 6]. The smaller skeletal muscle mass activated to perform certain activities does not achieve the same whole-body metabolic rate. Metabolic equivalents (METS) are often used to express the energy costs of PA as multiples of resting metabolic rate (RMR) [7]. However, the conventional MET value (oxygen uptake of 3.5 mL kg^−1^ min^−1^) is not applicable for persons with a disability, as disuse/paralysis results in atrophy of leg fat-free mass (FFM) [8, 9]. RMR is influenced by FFM [10], which explains why RMR is reduced in persons with disabilities who use wheelchairs compared to adults without disabilities [11]. For example, commonly used equations to predict RMR in persons with spinal cord injury (SCI) overestimate measured requirements by 5–32% [12]. Considering RMR is the largest component of total daily energy expenditure (TDEE) (up to 80% for sedentary individuals [13]), error in the prediction of this component using existing algorithms for persons without disabilities can have profound implications for accurately predicting TDEE. Consequently, approaches that solely measure physical activity energy expenditure (PAEE) might have greater utility, particularly as this is the most malleable component of TDEE.

### Measurement Methods

The PA monitoring field is evolving at a rapid pace. However, the development of validated self-report and objective tools to quantify PA/EE in persons who use wheelchairs remains relatively under researched. It is not always feasible to use criterion methods (i.e. indirect calorimetry, observation, doubly labelled water) to measure free-living PA/EE, as these techniques require expensive/sophisticated equipment or are impractical for use outside of the laboratory. Therefore, this review provides an overview of the predominant methods of measuring PA/EE in persons who use wheelchairs. Specifically, we describe and review the different self-report and objective tools currently available whilst also considering their potential strengths and limitations.

#### Self-report Measures

Until recently, the quantification of free-living PA in persons who use wheelchairs had been reliant on outputs from self-report measures [14, 15]. Self-report questionnaires offer researchers an inexpensive and easy-to-administer method of measuring PA. However, these methods are reliant upon the accuracy of the participants’ memory and recall. Furthermore, it has been suggested that self-report measures fail to adequately quantify the lower end of the PA continuum [16, 17], suffer from floor effects (lowest score is too high for inactive respondents) and participant over-reporting [18]. Besides these general limitations, specific issues pertaining to the administration of the three predominant questionnaires (Table 1) used to predict components of PA in this population are discussed below. It is noteworthy that not all were developed and/or validated for persons who use wheelchairs.

**Table 1:** Characteristics of questionnaires used previously to measure components of PA in persons who use wheelchairs

	PADS [19]	PASIPD [22]	PARA-SCI [27]
Items/administration/duration	46-item semi-structured interview or self-administered questionnaire (20–30 min)	13-item self-administered questionnaire (~15 min)	Semi-structured interview whereby a series of flow charts help the interviewer guide the participants through 8 periods of the day (20–45 min)
Timeframe	7 days	7 days	3 days
Dimensions	1. Exercise 2. LTPA 3. General activity 4. Therapy 5. Employment/school 6. Wheelchair use	1. Home repair/gardening 2. Housework 3. Vigorous sport 4. Moderate sport 5. Occupation	1. LTPA 2. ADL
Outcome	Score is based on the time respondents spend doing the activities multiplied by an intensity rating of that activity. Each activity has an assigned weighting (Aerobic = .3, strength = .2 and flexibility = .1). Higher scores represent more activity and negative scores can be achieved through sedentary behaviour	Number of days per week and hours per day of participation in above dimensions. Intensity of activity is established by multiplying the average hours per day for each item by a standard MET value (MET-h/day)	The mean number of minutes per day spent in mild, moderate, and heavy intensity LTPA and ADL. Scores may be summed to generate total accumulated PA (min/day)

*ADL* activities of daily living, *LTPA* leisure time physical activity, *PA* physical activity, *PADS* Physical Activity and Disability Survey*, PARA-SCI* Physical Activity Recall Assessment for People with Spinal Cord Injury, *PASIPD* Physical Activity Scale for Individuals with Physical Disabilities

The Physical Activity and Disability Survey (PADS) [19] was one of the first questionnaires developed but was validated for participants with a wide range of disabilities ranging from stroke to type-2 diabetes, and subsequently, a revised version (PADS-R) in persons with neurological conditions [20, 21]. Therefore, it could be argued that the content of the PADS fails to capture activities specific to the lifestyle of persons that use wheelchairs. The Physical Activity Scale for Individuals with Physical Disabilities (PASIPD) [22] was adapted from the Physical Activity Scale for the Elderly (PASE) and follows a similar format to that of the International Physical Activity Questionnaire (IPAQ) [23]. Despite being developed in people with both visual and auditory disabilities, its implementation in people with locomotor impairment and SCI means it could be considered sensitive to persons who use wheelchairs. However, only the Physical Activity Recall Assessment for People with Spinal Cord Injury (PARA-SCI) was specifically developed and evaluated for people with SCI.

##### Questionnaire Administration

A distinguishing feature between the three questionnaires is the resource demand to complete each tool (Table 1). The PARA-SCI was designed as an interview-based questionnaire that collects rich behavioural data. Thus, the PARA-SCI is resource intensive because it was developed, as a research tool, to be used in epidemiological studies. For example, it can take between 20–45 min to complete, the cost of the interviewer needs to be considered and there is considerable participant demand. Ullrich et al. [24] also suggested that the use of the PARA-SCI might have limited application for other investigators, besides the developers, due to the exclusion of subjective appraisals and the technical complexity of interview administration. These limitations were acknowledged by the authors who subsequently developed a new questionnaire to address these limitations. The Leisure Time Physical Activity Questionnaire for People with Spinal Cord Injury (LTPAQ-SCI) [25] is a brief (5 min) self-report questionnaire specifically designed for persons with SCI that measures minutes of mild, moderate and heavy-intensity leisure time physical activity (LTPA) performed over the previous 7 days but is not capable of measuring other activities of daily living.

##### Reliability and Validity

The test-retest reliability of the three questionnaires has been examined; however, the PADS has had no reliability studies conducted in persons who use wheelchairs. Therefore, it remains unclear whether the PADS can be reliably used as a measure of physical activity behaviour in this population. A test-retest reliability correlation of .77 was established for the PASIPD in a study of 45 adult patients with a range of disabilities, but these patients did not use wheelchairs [26]. The PARA-SCI is the only instrument tested for reliability in a sample solely consisting of persons who use wheelchairs. To establish the test-retest reliability of the PARA-SCI, 102 people with SCI completed the instrument on two separate occasions a week apart [27]. Intra-class correlations revealed good test-retest reliability for total cumulative activity (.79). However, moderate-intensity (LTPA) and heavy-intensity (lifestyle activity) demonstrated poor levels of reliability (ICC = .45 and .56, respectively).

Establishing the validity of questionnaires is important to ensure that the tool effectively measures what it intends to (i.e. the activity of persons that use a wheelchair). Manns and colleagues [28] revealed a significant moderate relationship between scores on the PADS and V̇O_2_ max (*r* = 0.45). Likewise, comparison of scores from the PASIPD with indicators of physical capacity revealed weak to moderate relationships (V̇O_2_ max; *r* = 0.25, manual muscle test; *r* = 0.35) [29]. However, we contend that equating self-reported PA to physical capacity, rather than a criterion measure of PA, may not be the most appropriate way to ascertain concurrent validity. Measures of physical capacity can be related to numerous variables beyond the users’ PA level.

Results from validity studies indicate that of the three questionnaires, the PARA-SCI has the strongest relationships with criterion measures. During the development and evaluation of the PARA-SCI [27], criterion (V̇O_2_ reserve) values displayed a very large correlation with cumulative (LTPA plus lifestyle) activity data (*r* = 0.79). When data was coded for intensity of activity, large to very large positive correlations were seen for moderate-intensity (*r* = 0.63) and heavy-intensity (*r* = 0.88) activity. However, this relationship was weak and non-significant for low-intensity activities (*r* = 0.27) and consequently, the PARA-SCI under-reported time spent doing activities of low intensity by 10%. Therefore, although these findings indicate some evidence of convergent validity, the results also highlight limitations of self-report measures.

##### Measuring Intensity

A distinguishing feature between the three disability questionnaires is how they gather information pertaining to the intensity of activity conducted. Evidence from adults without disabilities would suggest superior reductions in mortality risk with vigorous-intensity PA in comparison to light-to-moderate intensity PA [30–32]. Therefore, failure to consider individual differences in PA intensity makes it difficult to detect relationships between lifestyle activities and health outcomes [27]. The PADS employs a single item to examine the overall intensity of structured activity but does not assess the intensity of leisure time activities. A fundamental limitation of the PASIPD is the use of standard MET values as a measure of activity intensity regardless of the participant’s type of disability. If MET values are to be used, it will be necessary to develop a new empirically based supplement to the compendium of physical activity appropriate for persons that use wheelchairs [33]. The inability of the PASIPD and PADS to effectively measure activity intensity prompted the development of the PARA-SCI. Subsequently, the authors of the PARA-SCI conducted a systematic process to develop definitions of three different exercise intensities (i.e. mild, moderate and heavy) specifically for people with SCI [27]. The empirical development of intensity-based definitions suggests the PARA-SCI may be the most effective self-report questionnaire at measuring the intensity of PA in persons with SCI. However, it should be noted that even with such a rigorous development of intensity definitions, the PARA-SCI is still dependent upon the accurate recall of behaviour. Research has also challenged the use of psychophysiological indexes as a measure of perceived exertion in persons with SCI [34]. This could have implications for the prediction of activity intensity using self-report measures in persons with disabilities, which could be influenced by secondary conditions such as chronic pain and discomfort, coupled with the inability to engage large muscle groups in constant rhythmic activities.

Objective sensors overcome many of the shortcomings of self-report methods, predominantly by removing the subjective recall element. The next section will discuss the use of these objective sensors in wheelchair users.

#### Accelerometers

Accelerometers or movement sensors report their outcomes in ‘activity counts’ per unit time or epoch, which are the product of the frequency and intensity of movement. Accelerometers are therefore capable of providing temporal information about specific variables such as the total amount, frequency and duration of PA [35]. They can also monitor the accumulation of moderate-to-vigorous intensity PA (MVPA) and/or sedentary behaviour thanks to the development of population-specific cut-points for activity counts per minute. Despite enormous differences in signal processing and internal components, all accelerometers have similar fundamental properties defined by accuracy, precision, range and sensitivity and should be compared against criterion measurements to demonstrate validity [36]. Monitors have been compared to a selection of criterion laboratory measurements in persons that use wheelchairs: oxygen uptake (V̇O_2_) [37–39], EE [40, 41] and PAEE [42, 43] measured by indirect calorimetry (Table 2). Studies have utilised different commercial monitors, worn at various locations, validated using diverse activity protocols including propulsion on a wheelchair-adapted treadmill, wheelchair ergometer or over ground, arm-crank ergometry (ACE) and various activities of daily living. Two fundamentally different varieties of accelerometers are widely used in PA research, uniaxial and, increasingly, tri-axial. Uniaxial accelerometers register movement in the vertical axis only, whereas tri-axial accelerometers register movement in the anteroposterior (X), mediolateral (Y) and vertical (Z) axes. In keeping with pooled data from a systematic review of laboratory and free-living validation studies in adults without disabilities [44], it appears that the greater sensitivity of the tri-axial accelerometer leads to a better prediction of EE than uniaxial accelerometers in persons who use wheelchairs (Table 2).

**Table 2:** Summary of the accuracy of accelerometers worn on various anatomical locations and wheelchair during laboratory protocols. Comparison to criterion measures of oxygen uptake, energy expenditure and physical activity energy expenditure

Study	Sample^a^	Criterion measure	Activity protocol	Device/outputs	Anatomical location	Results
Garcia-Masso et al. [38]	20 SCI (T4-S1)	V̇O_2_ (COSMED K4b^2^)	Ten activities which included ADL, transfers, ACE and propulsion that covered a wide range of exercise intensities.	GT3X (36 features extracted from the second-by-second acceleration signals were used as independent variables)	Non-dominant wrist	*r* = 0.86, MSE = 4.98 ml kg^−1^ min^−1^
					Dominant wrist	*r* = 0.86, MSE = 5.16 ml kg^−1^ min^−1^
					Chest	*r* = 0.68, MSE = 10.41 ml kg^−1^ min^−1^
					Waist	*r* = 0.67, MSE = 10.61 ml^.^kg^−1.^min^−1^
Learmonth et al. [39]	24 (9 F). SCI (*n* = 10), SB (*n* = 5), MS (*n* = 4), AMP (*n* = 2), CP (*n* = 1), Other (*n* = 2)	V̇O_2_ (COSMED K4b^2^)	Three wheelchair propulsion speeds (1.5, 3.0 and 4.5 mph) on a WT	PAC from GT3X ACC	Right wrist	*r* = 0.95
					Left wrist	*r* = 0.93
					Combined	*r* = 0.94
Washburn & Copay, [37]	21 (9 F). SCI (*n* = 11), SB (*n* = 7), other (*n* = 3)	V̇O_2_ (Aerosport TEEM 1000)	Three timed pushes (slower than normal, normal, and faster than normal) over a rectangular indoor course	PAC from a CSA uniaxial ACC	Left wrist	*r* = 0.67, SEE = 4.99 ml^.^kg^−1.^min^−1^
					Right wrist	*r* = 0.52, SEE = 5.71 ml^.^kg^−1.^min^−1^
Hiremath & Ding, [54]	24 SCI (5 F) (T3-L4)	IC EE (COSMED K4b^2^)	Resting and three activity routines; propulsion (performed on a WERG and flat tiled surface), ACE (20–40) and deskwork.	PAC from a RT3 tri-axial ACC and participant demographics	Waist	*r* = 0.66, SEE = 1.38 kcal^.^min^−1^. EE estimation errors ranged from 12.9 – 183.4%
					Upper left arm (general equation)	*r* = 0.83, SEE = 1.02 kcal · min^−1^. EE estimation errors ranged from 14.1% – 113.7%
					Upper left arm (activity specific equations)	*r* ranged from 0.63 (deskwork) to 0.91 (propulsion). EE estimation error ↓ to between 12.2 and 38.1%
					Combined (Waist and upper arm)	*r* = 0.84, SEE = 1.00 kcal^.^min^−1^. EE estimation errors ranged from 14.1% – 116.9%
Kiuchi et al. [41]	6 SCI (C6 – T9)	IC EE (AR-1 Type-4)	Propulsion at three continuous speeds on a WT that elicited an RPE of 9 (2.5–3 km/h), 11 (3.5–4.0 km/h) and 13 (4.5–5.0 km/h)	Tri-axial ACC with gyro sensor. EE was predicted by incorporating acceleration, angular velocity and participant demographics	Left wrist	*r* = 0.93
					Right wrist	*r* = 0.82
					Left upper arm	*r* = 0.87
					Right upper arm	*r* = 0.93
Hiremath et al. [60]^b^	45 SCI (6 F) (C5 – L5)	IC EE (COSMED K4b^2^)	Participants performed 10 activities from a list that included a range of activities and exercises of differing intensities	Gyroscope-based wheel rotation monitor (G-WRM) and tri-axial accelerometer; The Physical Activity Monitoring System (PAMS)	PAMS-arm	ICC = 0.82 (95% CI; 0.79 – 0.85) M ± E = 9.82 ± 37.03%, MAE = 29.04%
					PAMS-wrist	ICC = 0.89 (95% CI; 0.87 – 0.91) M ± E = 5.65 ± 32.61%, MAE = 25.19%
Nightingale et al. [42]	15 (3 F). SCI (*n* = 9), SB (*n* = 2), Other (*n* = 4)	IC PAEE (COSMED K4b^2^)	Five activities including deskwork and wheelchair propulsion at various velocities around an outdoor athletics track.	PAC from GT3X + ACC	Right wrist	*r* = 0.93, SEE = 0.80 kcal^.^min^−1^. Absolute bias ± 95% LoA: 0.0 ± 1.55 kcal^.^min^−1^
					Right upper arm	*r* = 0.87, SEE = 1.05 kcal^.^min^−1^. Absolute bias ± 95% LoA: 0.0 ± 2.03 kcal^.^min^−1^
					Waist	*r* = 0.73, SEE = 1.45 kcal^.^min^−1^. Absolute bias ± 95% LoA: 0.0 ± 2.82 kcal^.^min^−1^
Nightingale et al. [43]	17. SCI (*n* = 10), SB (*n* = 3), CP (*n* = 1), AMP (*n* = 1), Other (*n* = 2).	IC PAEE (TrueOne 2400, ParvoMedics)	A wheelchair propulsion protocol across a range of treadmill velocities (3 – 7 km/h and gradients (1 – 3%) including load carriage (+8% body mass) and a folding clothes task	PAC from GT3X+ ACC	Right Wrist	*r* = 0.82, SEE = 0.91 kcal^.^min^−1^. MAE = 0.69 kcal^.^min^−1^ (33.0%)
					Right Upper Arm	*r* = 0.68, SEE = 1.16 kcal^.^min^−1^. MAE = 0.86 kcal^.^min^−1^ (35.3%)
				Raw acceleration (g · s^−1^) from GENEActiv ACC	Right Wrist	*r* = 0.88, SEE = 0.75 kcal^.^min^−1^. MAE = 0.59 kcal^.^min^−1^ (21.0%)
					Right Upper Arm	*r* = 0.87, SEE = 0.77 kcal^.^min^−1^. MAE = 0.58 kcal^.^min^−1^ (20.4%)

*AB* able-bodied, *ACC* accelerometer, *ACE* arm crank ergometry, *ADL* activities of daily living, *AMP* amputee, *CP* cerebral palsy, *CSA* computer science applications, *EE* energy expenditure, *IC* indirect calorimetry, *LoA* limits of agreement, *MAE* mean absolute error, *MS* multiple sclerosis, *MSE* mean square error, *M ± E* mean signed error, *PAC* physical activity counts, *PAEE* physical activity energy expenditure, *SB* Spina Bifida, *SCI* spinal cord injury, *SEE* standard error of estimate, *ULAM* upper limb activity monitor, *V̇O*_*2*_ oxygen uptake, *WERG* wheelchair ergometer, *WT* wheelchair treadmill

^a^All male participants unless stated otherwise

^b^Note to avoid confusion and make interpretation easier, + or − before M ± E statistics has been switched to reflect whether prediction method over or under predicted EE

##### Monitors Attached to the Wheelchair

Researchers have explored attaching a custom data logger [45] or biaxial [46] and tri-axial [47] accelerometers onto the wheels of a wheelchair. Other preliminary research has simply attached a smartphone (containing a gyroscope and accelerometer) onto the armrest of a wheelchair [48, 49]. Considering the exponential growth of smartphone ownership [50], this later approach in particular can widely be used to capture certain mobility characteristics such as average speed and distance travelled, functioning in a similar manner to pedometers in persons who do not use wheelchairs. However, despite these approaches being relatively unobtrusive, they are unable to quantify the intensity of activities being performed and are limited in deriving accurate EE estimates. Conger et al. [51] tried to address this limitation by using a PowerTap Hub attached to the wheel of a wheelchair. The measured hand rim propulsion power explained 48% of the variance in predicting criterion EE. The authors revealed three significant prediction models from this laboratory protocol, with model 3 (incorporating power, speed and heart rate) explaining the greatest variance (87%). Seemingly, the incorporation of a physiological signal significantly improved the prediction of EE.

However, we propose that a device attached to the wheelchair cannot distinguish between self or assisted propulsion, certainly not without complex analyses of the raw acceleration outputs [52], and are unable to quantify activity out of the wheelchair. Further, it is common for persons who use wheelchairs to have different chairs to participate in various sports or undertake ACE as a mode of exercise. Therefore, a single device attached to a wheelchair will fail to capture moderate-to-vigorous-intensity activity in structured exercise, likely to contribute a large proportion towards TDEE. Moreover, if a person uses a power-assisted wheelchair, signals from devices attached to the chair will provide erroneous measurements regarding upper body PAEE. These limitations need to be considered when using this approach to predict free-living PA/EE in persons that use wheelchairs.

##### Body-Borne Accelerometers

Waist-mounted single-sensor devices, positioned within close proximity to an individual’s centre of mass, have been the mainstay of activity monitoring in cohorts without physical disabilities. Single units worn on the waist can be limited for certain types of upright behaviours that have a low ambulatory component and may involve upper-body work [53]. The measurement error of waist-mounted devices is generally related to the inability to detect arm movements as well as static work (e.g. lifting, pushing, carrying loads). With movement of persons that use wheelchairs predominantly restricted to the upper-body, it is unsurprising that stronger correlations between accelerometer outputs and criterion measurements were reported for devices worn on the upper arm and wrist, *r* = 0.83–0.93 and *r* = 0.52–0.93, respectively (Table 2). While two studies [37, 41] have found differences in the strength of correlations between the left and right wrist, these discrepancies could be due to hand dominance or the specific asymmetry of the activities performed in these studies. The predominance of research, however, suggests little to no difference between dominant and non-dominant wrists [38, 39], suggesting freedom/flexibility in selecting either wrist to predict PA/EE in this population.

Combining data from two anatomical locations seemingly does not yield substantial improvements in the strength of correlations or EE estimation error [39, 54]. In some research and development laboratories, accelerometers have been arranged in parallel arrays and positioned at various anatomical locations to monitor the types of activity being performed by postural identification. Such prototype PA monitors were developed to primarily target specific population groups during rehabilitation, including amputees [55] or inpatients with SCI [56]. Devices with multiple arrays have shown good specificity (92%), agreement (92%) and sensitivity (87%) for the detection of wheelchair propulsion in observational studies [57]. Yet, even when worn for a relatively short period of time, participants self-reported moderate burden [58]. These monitors are relatively obtrusive and, due to reduced memory capacity and battery life, are restricted to short monitoring durations (<48 h). This is not in keeping with current end user requirements of PA monitors. Multi-site prototype arrays are also not typically available outside of the developing laboratory, making validation by other researchers challenging.

A simpler set-up, the Physical Activity Monitoring System (PAMS) [59], which incorporates a gyroscope-based wheel rotation monitor (G-WRM) and one tri-axial accelerometer attached to the arm or wrist, overcomes the shortcomings of accelerometers attached to the wheelchair alone. When this approach was recently evaluated using a robust laboratory protocol and home-based follow-up session, both the PAMS-arm and PAMS-wrist estimated EE with small biases (M ± E < 10%) [60]. Yet, MAE for predicting EE in persons that use a wheelchair remained elevated (>25%). Kooijmans et al. [61] also assessed the utility of a tri-axial accelerometer (GT3X+) attached to the wrist and spokes of a wheelchair. However, rater observations reported less agreement (85%) and specificity (83%) for wheelchair propulsion than using multiple-arrays [57]. Whilst less burdensome, disagreement between GT3X+ (Actigraph, Pensacola, FL) outputs and observers was greatest for propulsion on a slope and being pushed whilst making excessive arm movements. Therefore, it is likely that physiological signals, such as heart rate, should be incorporated into the prediction of EE to improve accuracy.

#### Heart Rate

Heart rate (HR) is useful as a physiological variable as it increases linearly and proportionately with exercise intensity and thus oxygen uptake [36], at least in individuals without disabilities. Keytel et al. [62] concluded that EE can be accurately predicted from HR after adjusting for age, sex, body mass and fitness. However, during lower intensity PA, there is a weak relationship between HR and EE [63]. This is most likely due to small postural changes causing alterations in stroke volume, or that HR during low intensity PA is affected by external factors such as psychological stress, stimulants, ambient temperature, dehydration and illness [64]. There are a number of ways to use HR data to predict EE, one of the most promising being the FLEX-HR method [65], which has previously been used in persons with SCI [66, 67]. Despite recent research into the use of various HR indices [68] and artificial neural networks [69] in the prediction of V̇O_2_ in individuals with SCI, it is clear that the accurate prediction of EE using HR is heavily reliant on individual calibration. Hayes et al. [67] found that the variance in measured EE was considerably improved using an individual calibration (55%) compared to HR alone (8.5%) during five activities of daily living in thirteen individuals with SCI. Considering the type of activities performed, the large variations in cardiovascular fitness and cardiovascular responses to exercise stress persons who use wheelchairs, individual specific HR-EE relationships are necessary for the accurate prediction of EE using HR. This consideration is perhaps even more important for persons with considerable functional impairment or various disability aetiologies that may disrupt the autonomic nervous system, such as high-level SCI (>T6).

#### Multi-Sensor Devices

New multi-sensor technologies, which include the combination of physiological parameters and accelerometry, have great potential for increased accuracy in assessing EE as they incorporate and minimise the strengths and weaknesses of physiological signals and accelerometry alone. The use of multi-sensor devices has mostly been limited to laboratory based validation of the SenseWear® Armband (SWA) (BodyMedia Inc., Pittsburgh, PA), which is worn on the upper arm, a preferential anatomical location for the prediction of EE in persons that use wheelchairs (Table 2). More detailed components and specifications of this activity monitor have been described elsewhere [36]. It is clear that the proprietary manufacturer’s algorithms intrinsic to the SWA device are not appropriate to predict EE in persons that use wheelchairs, with ICCs < 0.64 [40, 70, 71]. The overestimation of EE by the SWA manufacturer’s model is likely due to the movements typically performed by persons that use wheelchairs (e.g. wheelchair propulsion and ACE) not being included in predefined activity categories. Hence, such activities are misclassified into more strenuous types of PA.

Researchers have developed new EE prediction models (SCI general and activity specific) for the SWA device that have been cross-validated [40, 71]. Where MAE statistics are available [40, 70, 71] weighted means were calculated, with the SCI general (22.7%) and activity specific (18.2%) models performing significantly better than the manufacturer’s model (54.4%). Whilst these findings provide encouragement for the use of the SWA in persons that use wheelchairs with new prediction models, Conger et al. [72] noticed that even when using the SCI general model, the SWA tended to overestimate EE (27 to 43%), whereas a wrist-mounted accelerometer more accurately predicted EE (9 to 25%) during wheelchair prolusion (Table 3). It is noteworthy that the SWA utilizes upwards of twenty possible output parameters, including heat flux, galvanic skin response and temperature to predict EE. Individuals with high level SCI (>T6) experience impaired thermoregulatory function (reduced sweating response and inability to dilate superficial vasculature [73]), which might intrinsically effect the error when using SWA in this population. Unfortunately, the acquisition of the company BodyMedia by Jawbone in 2013 resulted in the device being taken off the market and cessation of all BodyMedia web applications. Despite considerable improvements in EE prediction error it seems the future use of this technology is limited.

**Table 3:** Summary of the accuracy of multi-sensor devices in persons who use wheelchairs during laboratory protocols

Study	Sample^a^	Criterion measure	Activity protocol	Device and location	Results
Conger et al*.*[109]	14 (3 F). SCI (*n* = 7) SB (*n* = 4) AMP (*n* = 2) Other (*n* = 1)	IC EE (Oxycon Mobile)	Five different wheeling activities. Propulsion on a level surface (4.5, 5.5 & 6.5 km/h), wheeling on a rubberised 400 m track (5.5 km/hr) & wheeling on a sidewalk course at a S-S speed	Actical on right wrist	No sig. differences between criterion method and Actical EE (±9 – 25%)
				SWA on right upper arm	Sig. overestimated EE during wheelchair propulsion (+30 - 80%)
				SWA using SCI general model (Hiremath and Ding, [70])	↓ EE prediction error (+27-43%), yet, this was still elevated during higher intensity activities
Hiremath & Ding [40]	24 SCI (5 F) (T3-L4)	IC EE (COSMED K4b^2^)	Resting and three activity routines; propulsion (performed on a WERG and flat tiled surface), ACE (20-40) and deskwork	Estimated EE from RT3 tri-axial ACC worn on the waist	R_S_ = 0.72 for all activities (↓ for propulsion; R_S_ = 0.44, ↑ for deskwork; R_S_ = 0.66). EE estimation errors ranged from 22.0 to 52.8%. Poor ICCs 0.64
				Estimated EE from SWA worn on the upper arm (manufacturer’s model)	R_S_ = 0.84 for all activities (↓ for deskwork; R_S_ = 0.65, ↑ for propulsion; R_S_ = 0.76). EE estimation errors ranged from 24.4 to 125.8%. Poor ICCs 0.62. Neither device is an appropriate tool for quantifying EE (<0.75)
Hiremath et al*.* [70]^c^	45 (8 F) (C4–L4)	IC EE (COSMED K4b^2^)		Estimated EE from SWA worn on the upper arm (manufacturer’s model)	ICC = 0.64 (95% CI; 0.57–0.70) M ± E = 51.5 ± 31.6% MAE = 2.0 kcal · min^-1^ (59.2%)
				Estimated EE from SWA worn on the upper arm (SCI general model)	ICC = 0.72 (95% CI; 0.66 – 0.77) M ± E = -10.4 ± 11.8% MAE = 0.9 kcal · min^-1^ (24.7%)
				Estimated EE from SWA worn on the upper arm (activity-specific model)	ICC = 0.86 (95% CI; 0.82–0.88) M ± E = −9.6 ± 10.9% MAE = 0.6 kcal · min^-1^ (16.8%)
Tsang et al. [71]^c^	45 SCI^b^ (6 F) (C5 – L5)	IC EE (COSMED K4b^2^)	Participants performed 10 activities from a list that included a range of activities and exercise of differing intensities	Estimated EE from SWA worn on the upper arm (manufacturer’smodel)	ICC = 0.62 (95% CI; 0.16 – 0.81) M ± E = 39.6 ± 37.8% MAE = 43.3 ± 33.5%
				Estimated EE from SWA worn on the upper arm (SCI general model)	ICC = 0.86 (95% CI; 0.82 – 0.89) M ± E = 2.8 ± 26.1% MAE = 20.6 ± 16.2%
				Estimated EE from SWA worn on the upper arm (activity-specific model)	ICC = 0.83 (95% CI; 0.79 – 0.87) M ± E = 4.8 ± 25.4% MAE = 19.6 ± 16.8%
Nightingale et al*.* [75]	15. SCI (*n* = 8), SB (*n* = 3), CP (*n* = 1), AMP (*n* = 1), Other (*n* = 2)	IC PAEE (TrueOne 2400, ParvoMedics)	A wheelchair propulsion protocol across a range of treadmill velocities (3–7 km/h and gradients (1–3%) including load carriage (+8% body mass) and a folding clothes task	Actiheart^TM^ using manufacturers proprietary algorithms	*r* = 0.76 (*P* < 0.01), SEE = 1.07 kcal · min^-1^ mean bias ± 95% LoA = 0.51 ± 3.75 kcal · min^-1^ MAE = 1.35 kcal · min^-1^ (51.4%)
				Actiheart^TM^ using individual heart rate calibration	*r* = 0.95 (*P* < 0.01), SEE = 0.49 kcal · min^-1^ mean bias ± 95% LoA = - 0.22 ± 0.96 kcal · min^-1^ MAE = 0.39 kcal · min^-1^ (16.8%)

*ACC* accelerometers, *ACE* arm crank ergometry, AHR Actiheart^TM^, *AMP* amputee, *CP* cerebral palsy, *EE* energy expenditure, *IC* indirect calorimetry, *LoA* limits of agreement, *MAE* mean absolute error, *PAEE* physical activity energy expenditure, *SB* Spina Bifida, *SCI* spinal cord injury, SEE standard error of estimate, SWA SenseWear® Armband, S-S self-selected, *WERG* wheelchair ergometer

^a^All male participants unless stated otherwise

^b^Independent sample of participants to previous Hiremath et al. [70] trial in table

^c^ Note to avoid confusion and make interpretation easier direction, + or − before M ± E statistics has been switched to reflect whether prediction method over or under predicts EE

The Actiheart (Cambridge Neurotechnology Ltd, Papworth, UK) integrates an accelerometer and HR monitor into a single-piece movement monitor. The Actiheart (AHR) unit has been described in detail previously [4], along with the detailed branched modelling technique it utilises to estimate PAEE through the combination of HR and accelerometer counts [74]. Previous work from our research group [75] has assessed the performance of this device in a controlled-laboratory environment with a heterogeneous sample of persons who use wheelchairs. Across all activities considerable mean absolute error (MAE) was reported (51.4%) using the manufacturer’s proprietary algorithms to predict PAEE. By using an incremental ACE test, which permitted an individual HR-EE relationship similar to that performed by Hayes et al [67], individual calibration was incorporated and MAE was considerably reduced to 16.8% across all activities. Individual calibration has also been shown to improve the prediction of EE estimations using this device in free-living [76, 77] and laboratory settings [78] in adults without disabilities during walking and running. The sizeable improvement in EE prediction error in persons who use wheelchairs with individual calibration may be due to a larger degree of individual variance in cardiovascular function and responses to exercise in this population. Consequently, individual calibration of this monitor is of upmost importance for the accurate prediction of PAEE in persons that use wheelchairs. Furthermore, incorporating individually calibrated HR and acceleration data better captures the differing energy costs of bespoke activities, despite similar acceleration profiles, such as wheelchair propulsion up a gradient or with additional load (e.g. shopping) [75].

### Prediction Accuracy of Methodologies in Free Living Environments

The majority of PA/EE validation research in this population has been performed in a controlled-laboratory environment but there is a paucity of free-living studies (Table 4) primarily due to the practical difficulties or expense associated with ‘gold standard’ EE measurement (DLW). This method is not without limitations; for example minimal information regarding frequency, duration or intensity of activity can be obtained [79]. Furthermore, the estimation of EE is based on the assumption of a mean respiratory exchange ratio (RER) of 0.85, indicative of a standard western diet [80]. Yet, carbohydrate and fat oxidation has been shown to be altered with arm compared to leg exercise [81] and in paraplegics compared to non-disabled controls [82]. These factors may lead to an increased RER in persons that use wheelchairs, which could violate the assumptions used in the prediction of EE via the DLW technique.

**Table 4:** Summary of free-living energy expenditure estimation studies in persons who use wheelchairs

Study	Sample^a^	Reference standard	Monitoring duration	Method	Results
Tanhoffer et al. [66]	14 SCI (1 F). (C4–T12)	TDEE (DLW)	14 days	SenseWear worn on the upper arm (manufacturer’s model)	*R* ^2^ = 0.65 (*P* < 0.001) Mean bias ± 95% LoA = 382 ± 898 kcal · day^-1^ (16% over-prediction)
				FLEX-HR	R^2^ = 0.68 (*P* = 0.001) Mean bias ± 95% LoA = -205 ± 655 kcal · day^-1^ (13% under-prediction)
				PARA-SCI	*R* ^2^ = 0.74 (*P* < 0.001) Mean bias ± 95% LoA = -133 ± 598 kcal · day^-1^ (6% over-prediction)
				PASIPD	*R* ^2^ = 0.53 (*P* = 0.003) Mean bias ± 95% LoA = - 12 ± 819 kcal · day^-1^ (1% over-prediction)
		PAEE (TDEE (measured by DLW) x 0.9) – RMR (IC)		SenseWear worn on the upper arm (manufacturer’s model)	*R* ^2^ = 0.16 (*P* = 0.159) Mean bias ± SD = -16 ± 1292 kcal · day^-1^ 3% under-prediction
				FLEX-HR	*R* ^2^ = 0.30 (*P* = 0.067) Mean bias ± SD = 22 ± 715 kcal · day^-1^ 3% over-prediction
				PARA-SCI	*R* ^2^ = 0.50 (*P* = 0.005) Mean bias ± 95% LoA = - 120 ± 537 kcal · day^-1^ 18% under-prediction
				PASIPD	*R* ^2^ = 0.13 (*P* = 0.198) Mean bias ± 95% LoA = − 11 ± 737 kcal · day^-1^ 3% under-prediction
Nightingale et al. [75]	8. SCI (*n* = 5), SB (*n* = 2), CP (*n* = 1)	PAEE (Estimated from a physical activity log, using the adapted PA compendium (Conger and Bassett, [6])	24 h	Actiheart^TM^ using manufacturers proprietary algorithms	*R* ^2^ = 0.16 (*P* = 0.24) SEE = 365 kcal · day^-1^
				Actiheart^TM^ using individual heart rate calibration	*R* ^2^ = 0.50 (*P* = 0.03) SEE = 269 kcal · day^-1^
Warms et al. [83]	50 (23 F) wheelchair users. Mixed aetiology of disabilities	Daily physical activity record scores	7 days	Activity counts from a tri-axial Actiwatch	*r* = 0.506 (*P* = 0.000)
				PASIPD	*r* = 0.267 (*P* = 0.67)

*CP* cerebral palsy, *DLW* doubly labelled water, *LoA* limits of agreement, *PA* physical activity, *PAEE* physical activity energy expenditure, *PARA-SCI* physical activity recall assessment for people with spinal cord injury, *PASIPD* physical activity scale for individuals with physical disabilities, *RMR* resting metabolic rate, SCI spinal cord injury, *TDEE* total daily energy expenditure

^a^All male participants unless stated otherwise

Irrespective of this, Tanhoffer et al. [66] compared four aforementioned prediction methods (SWA, FLEX-HR, PARA-SCI, PASIPD) to DLW during habitual routines over an extended 14-day period. The authors demonstrated that the two best prediction methods were PARA-SCI and FLEX-HR for both TDEE and PAEE (Table 4). The SWA and PASIPD both performed particularly poorly in the prediction of PAEE, displaying considerable random error as demonstrated by the large 95% limits of agreement. It is noteworthy that the SWA used the aforementioned error-prone manufacturer’s model [40, 70, 71] but could be improved with the SCI general EE prediction model developed by Hiremath et al. [70]. One limitation of the Tanhoffer et al [66] study is that the length of PA monitoring period for each prediction method varied compared to the criterion method. Total EE collected over a two-week period for the criterion DLW technique was divided by 14 to estimate mean TDEE. However, the objective measures (SWA and FLEX-HR) were only worn ≥12 h on two separate days and subjective measures, the PARA-SCI and PASIPD ask participants to recall the previous 3 and 7 days, respectively. This weakness in the experimental design means it is difficult to identify whether the error is intrinsic to each prediction method or simply an artefact of the comparison between different days or time-periods.

In the absence of other suitable criterion free-living methods, researchers have encouraged the simple evaluation of the agreement and disagreement between measures [16]. Previous studies have compared prediction methods to daily PA record scores over 7 days [83] and PAEE estimated from the adapted PA compendium over 24 h [75]. Again the PASIPD was poorly correlated with the reference standard, whereas outputs from a tri-axial Actiwatch demonstrated a stronger correlation, *r* = 0.51 [83]. Nightingale and colleagues [75] supported their earlier laboratory findings, demonstrating that the Actiheart™ with individual HR calibration explained more of the variance in free-living PAEE than using the Actiheart™ with proprietary algorithms. However, these analyses were only performed on a subsample of participants (*n* = 8) who had provided enough detailed information in PA logs to allow accurate estimation of PAEE using the adapted PA compendium for manual wheelchair users [6]. This compendium only describes the energy cost of 63 wheelchair activities compared to the 821 specific activities included in the updated version of the compendium of physical activities for adults without disabilities [84]. Consequently, coding of activities is less specific and accuracy of data is reliant on the quality of the self-report PA log. There are clear discrepancies between validating objective tools in a controlled-laboratory and free-living environments. Therefore, renewed efforts are required to validate measurement tools in both settings to determine convergent validity.

### Statistical Approaches, Analytical Considerations and Future Directions

The majority of studies found strong associations between criterion measurements and outputs from wearable devices. However, in some instances where results from Bland Altman methods are also available, considerable random error has been reported [42, 66]. Where devices have been validated over a wide range of activities, of various intensities (in keeping with best practice guidelines [85]) a stronger correlation coefficient is likely. Consequently we encourage researchers to conduct multi-trait multi-method approaches [86], such as Bland Altman methods to assess agreement [87] or report measurement error [88]. It is also important that authors are very clear about what error calculations have been performed and what error statistics are reported. Furthermore, it would be advisable for the wider academic community to produce a consensus statement addressing the clinical limits for PA/EE assessment error for devices used in this population.

It is possible that predicting EE/PAEE from linear regression equations may be too simple an approach to use in examining complex movements or behaviours [89]. The activity protocols adopted by laboratory validation studies cited here mostly focus around wheelchair propulsion of various velocities. It is important to characterise this behaviour, as it will likely make a significant contribution to TDEE in free-living conditions (similar to ambulation in adults without disabilities). But as push frequency increases to match higher velocities, so too will accelerometer outputs. Therefore, whilst it might be appropriate to use linear regression methods to quantify PAEE associated with wheelchair propulsion, this approach might misclassify other types of physical activity. This is highlighted by considerable increases in measurement error for sedentary or atypical movements such as folding clothes [38, 43]. Greater error in more frequently performed low-intensity or sedentary behaviours has potentially considerable implications for the accurate determination of free-living EE in persons who use wheelchairs. A more ecologically valid approach would be to develop regression models based on a smorgasbord of activities common in the everyday lives of persons who use wheelchairs. It is possible that, by giving more weight to everyday activities (i.e. household chores or work-based tasks), such regression models may reduce estimation error.

An alternative solution to regression models would be to use new data analysis methodologies [90], including hidden Markov models [91], artificial neural networks [92, 93] and classification trees [94], which use the rich information to classify certain activities and derive a more accurate estimate of EE [85]. To obtain such rich information, the shortest possible epoch (1 s) should be selected for activity monitor data collection [95], primarily to maximise the original PA related bio-signal being retained. Garcia-Masso et al. [96] recently developed and tested classification algorithms based on machine learning using accelerometers to identify specific activities performed by persons who use wheelchairs. This is encouraging since activity-specific EE algorithms developed for resting, wheelchair propulsion, arm-ergometry and deskwork can improve overall EE estimation [70, 97]. One important consideration that remains to be addressed is, how well objective measurement tools and associated algorithms capture elevated energy expenditure during recovery from MVPA (i.e. excess post-exercise oxygen consumption). It is conceivable that a physiological signal is required to accurately capture this information when acceleration signals post-exercise might be similar to resting values. Future research should consider; (i) applying and further developing new data analysis techniques, (ii) using more ecologically valid assessments that better resemble free-living conditions for persons that use a wheelchair and, (iii) evaluating the performance of EE prediction models during recovery after exercise (which contributes to TDEE).

Some of the principal limitations of previous validation studies are the relatively small sample sizes recruited, the mixed aetiologies for wheelchair use and, use of EE prediction algorithms without cross-validation. This is likely due to difficulties associated with recruiting from various disabled populations [98], and we encourage research groups to work collaboratively to recruit larger sample sizes. Using a diverse sample of participants and aetiologies for wheelchair use has been widely adopted [37, 39, 72, 83] and provides a robust model for the assessment of EE in the wider population of individuals who use wheelchairs, rather than a subgroup of that population. When the development of regression equations to predict EE and subsequent evaluation was conducted on the same sample of participants [37, 42], there is a tendency for the evaluation statistics to be biased and overly optimistic [88]. Cross-validation is necessary, whereby the validity of developed algorithms are assessed using an independent sample of participants [54, 70]. We advocate employing a leave-one-out cross validation analysis [99] which has been employed previously [38, 43]. This permits an ‘independent’ assessment of EE prediction algorithms, and is an optimal approach when participant recruitment is particularly challenging.

### Wearable Technology and Physical Activity Guidelines for Persons who use Wheelchairs

The American College of Sports Medicine (ACSM) have highlighted wearable technology as the top fitness trend for 2016 [100]. Available consumer devices (Apple Watch, Microsoft Band, Fitbit Charge HR) are becoming increasingly sophisticated, incorporating multi-sensor technologies and are worn on the most appropriate anatomical location (wrist) to predict EE in persons who use wheelchairs. Apple recently announced at its Annual Worldwide Developer’s Conference that they have developed fitness tracking algorithms specifically for persons who use wheelchairs. Such wearable devices have the potential to provide wheelchair users with physical activity feedback which is informative and motivating [101]. The feasibility of combining estimation methods should also be explored. Greater context regarding the location, type and purpose of physical activity behaviours are of huge importance in public health research. More detailed information may be achieved by combination of GPS and accelerometer outputs, especially when also incorporating self-report measures. This approach could help to understand specific personal and environmental barriers to exercise, which are numerous for persons who use wheelchairs [102].

It has been suggested that individuals with disabilities should strive to meet PA guidelines of 150 min of MVPA per week [103]. These general population guidelines were informed by epidemiological evidence, using questionnaires, which capture the amount of activity required above normal lifestyle activities. While minutes per week represent an easy target for people to understand and attain, only the PARA-SCI and multi-sensor devices can currently be used in persons who use wheelchairs to generate total accumulated MVPA per day/week. Discrepancies have been shown between self-reported and objectively measured PA [104, 105]. Consequently, a recent review of data collected with accurate multi-sensor devices in adults without disabilities has suggested that ~1000 min per week of MVPA is a more appropriate target [106]. To our knowledge, only one paper has attempted to establish MVPA cut-points for wrist worn accelerometer outputs in persons who use wheelchairs [107]. However, accelerometer outputs alone (without complex data processing techniques) cannot easily detect the resistance of various movements that have similar acceleration profiles i.e. arm-crank exercise at 70 revolutions per minute; with no resistance (light-intensity activity) vs. 40 W (likely MVPA). Therefore, deriving MVPA cut-points for single unit wrist/arm accelerometers might have limited applicability, as direct outputs are unable to differentiate the resistance of certain arm movements (thus activity intensity) common in the everyday lives of persons who use wheelchairs. As such, measuring activity intensity is of utmost importance to accurately estimate MVPA, above and beyond daily PAEE/EE. Improvements in measurement techniques that capture this specific variable would significantly help to inform specific PA guidelines for persons with chronic disabilities who use wheelchairs.

## Conclusions

**Fig. 1:**
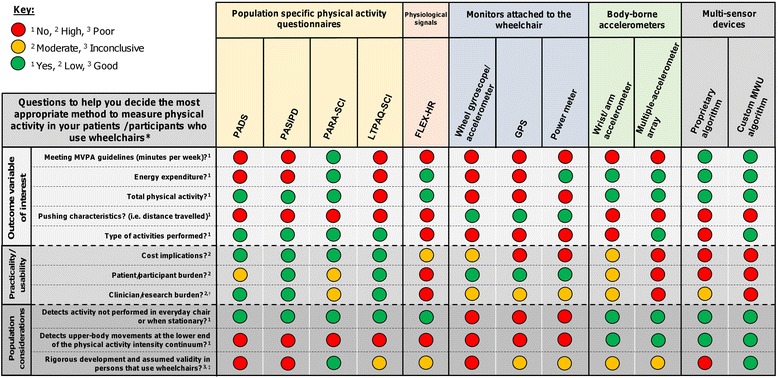
A guide for clinicians/researchers to help select the most suitable physical activity measurement tool in persons who use wheelchairs. *Asterisk* indicates researcher/clinician can decide which of these questions they consider most important. *Dagger* indicates taking into account the burden of tool administration and the complexities of data processing. *Double dagger* indicates based on the synthesis of evidence reported in this review. *Abbreviations*: *GPS* Global Positioning System, *LTPAQ-SCI* Leisure Time Physical Activity Questionnaire for People with Spinal Cord Injury, *MWU* manual wheelchair user, *PADS* Physical Activity and Disability Survey, *PARA-SCI* Physical Activity Recall Assessment for People with Spinal Cord Injury, *PASIPD* Physical Activity Scale for Individuals with Physical Disabilities

There is now a renewed impetus to translate progress in measuring PA in adults without disabilities to persons who use wheelchairs, with the techniques reviewed here (i.e. self-report, physiological signals, accelerometry and multi-sensor devices), displaying varying degrees of success. Currently, selecting a PA assessment tool to use in this population presents a challenging proposition for clinicians and researchers alike due to differing outcome variables of interest, practicality/usability of the tool and population specific considerations. To help guide decision-making, Fig. 1 was developed to provide a systematic evaluation of the strengths and limitations of the different measurement tools reported herein. The PARA-SCI has been extensively developed and is the most suitable self-report measure to predict time spent performing various intensity activities. This methodology also captures the type of activities being performed, categorised as either LTPA or activities of daily living, which provide useful behavioural information. Tri-axial accelerometers worn on the wrist or arm are well tolerated and relatively unobtrusive [108]. They offer a promising alternative to self-report methods for predicting PA/EE, particularly when combined with devices attached to the wheelchair or by incorporating complex data analysis methodologies. Multi-sensor devices, with algorithms developed specifically for the individual or generally for persons who use wheelchairs, demonstrate considerably improved error in the prediction of PA/EE during controlled laboratory protocols. It is possible that due to altered movement patterns and variations in metabolically active mass, predicting PA/EE in persons that use wheelchairs might be intrinsically more challenging. However, building on the current progress outlined in this review, we encourage the scientific community to rise to the challenge and provide innovative solutions to accurately predict free-living PA behaviours in this population. This is particularly important given the greater risk of non-communicable diseases, which are often associated with reduced activity, in persons with chronic physical disabilities who use wheelchairs.
